# The influence of observers on children’s conformity in moral judgment behavior

**DOI:** 10.3389/fpsyg.2024.1289292

**Published:** 2024-06-13

**Authors:** Yoonha Lee, Hyun-joo Song

**Affiliations:** Department of Psychology, Yonsei University, Seodaemun-gu, Seoul, Republic of Korea

**Keywords:** conformity, moral judgment, social influence, observers, reputation management

## Abstract

Children autonomously make sound moral judgments based on internal criteria, but they tend to make erroneous judgments in the presence of social influences, and the reasons for these errors are not well understood. Thus, the current research investigated how the presence of observers who can see and listen to 3-year-old children’s judgments but who do not present their opinions influences children’s conformity in moral judgment behavior. In Experiment 1, the children (*N* = 30) were presented with pictures depicting prosocial behaviors and asked whether the behaviors were acceptable. The children’s tendency to change their answers after hearing the counterintuitive opinions of informants was then measured. The results showed that the children’s moral judgments were more likely to conform to that of the group in the presence of observers. Experiment 2 aimed to determine the reason children were more likely to conform to a group when being watched by observers in Experiment 1. Children (*N* = 30) were randomly assigned to two conditions with different observer conditions as follows. Observers were either wearing headsets, indicating that they could not hear the children’s responses, or had them hanging around their necks, indicating that they could. The results showed that children’s conformity behavior depended on whether observers could hear what they were saying. The current findings are expected to help elucidate not only social factors that affect children’s moral judgments but also the developmental mechanism of an observer effect.

## 1 Introduction

Children can make good moral judgments on their own, but they often make erroneous judgments in the presence of social influences. A typical example of a social influence on children’s autonomous moral judgment is conformity. Despite their abilities to make correct moral decisions independently, 3-year-old children often conform to peer judgment after watching a video where a peer group deems a moral transgression (e.g., hitting a friend) as acceptable (Kim et al., 2016). Preschoolers generally view unfamiliar behaviors that lead to others crying as negative; however, their evaluations tend to be less harsh after witnessing informants rate these actions favorably (Li et al., 2019).

Although children’s group conformity in moral judgment behavior can have positive consequences, such as encouraging children to join a volunteer club, it can also lead to serious social issues like bullying. Furthermore, conforming to a group with morally incorrect opinions may hinder children’s moral development. Thus, it is crucial to study and discover which environmental factors influence children’s conformity in moral judgment behavior.

The extent of children’s conformity can be influenced by the specific context in which judgments are made. For instance, research has shown that 3- and 4-year-old first-generation Asian-American children displayed higher levels of conformity when an experimenter was present beside them, compared to when the experimenter was absent (Corriveau et al., 2013). Similarly, another study found that 4-year-old German children were more likely to conform to peers who provided incorrect information when their responses were spoken aloud, as opposed to quietly pointing with their fingers (Haun and Tomasello, 2011). In both cases, children exhibited a tendency to conform to the group’s judgments, even when they were incorrect, particularly in public settings.

A significant unanswered question pertains to the influence of passive observers who do not actively engage or express their opinions on children’s conformity in moral judgments. The mere presence of observers can impact human behavior and decision-making processes, even without explicit guidance or expression of opinions. Both young children and adults tend to display greater tolerance and prosocial behavior when they are being observed (Bereczkei et al., 2010; Leimgruber et al., 2012). In a study conducted by Engelmann et al. (2012), the frequency of 5-year-olds’ sharing stickers with friends increased when two peers were present, compared to when they were not, while the frequency of stealing stickers significantly decreased. These findings suggest that the desire to uphold one’s reputation and manage the impressions others have of oneself is a distinct human characteristic, and the mechanism driving this concern for reputation management develops in young children (Engelmann et al., 2012; Leimgruber et al., 2012). More recent evidence suggests that even 3-year-olds’ prosocial actions are influenced by the sense of being observed: 3-year-olds are more likely to act prosocially when exposed to images of eyes rather than images of flowers (Kelsey et al., 2018). This finding suggests that that reputation management emerges at least by the age of 3.

The phenomenon of the observer effect on conforming behavior may also stem from a social drive to appear comparable to others. In a study conducted by Haun et al. (2014), the impact of the observer effect on conformity behavior was examined in both 2-year-old children and apes closely resembling humans, such as chimpanzees and orangutans. The findings revealed that only human children demonstrated conformity behavior by adapting their actions based on the presence of peers who were observing them in a public setting. Furthermore, children exhibit a strong inclination toward individuals who share similarities with them, as opposed to those with contrasting attributes (Kinzler and Spelke, 2011). These preferences lead children to perceive themselves as akin to others, thus becoming a crucial mechanism for social affiliation. Consequently, the observer effect can be elucidated by the concepts of higher-order reputation, impression management, as well as social motives and sensitivities, including the aspiration to be perceived as akin to others.

While the influence of observers on children’s moral behaviors has been established, the impact on their moral judgments remains unexplored. Therefore, the objective of this experiment is to examine the role of an observer as a social factor that influences a child’s moral judgment. Specifically, the experiment aims to investigate whether the presence of observers leads to the child conforming to a group that makes an incorrect moral judgment. Furthermore, by exploring variations in children’s conformity behavior based on the identity of the observer, this experiment seeks to uncover the underlying mechanism of the observer effect.

## 2 Experiment 1

Experiment 1 aimed to explore whether 3-year-old Korean children conform to a group that makes incorrect judgments based on whether they are being observed by observers during moral decision-making. We chose to test 3-year-old children, guided by the results of previous studies (Corriveau et al., 2013 Kim et al., 2016), which demonstrated an elevation in their conformity behavior among 3-year-olds in public settings.

The experiment hypothesized that if children experience increased social pressure from observers who can witness their own judgments and the judgments of others, they would display higher levels of group conformity when observers are present compared to when only the experimenter is present. However, if children are not influenced by the presence of observers, it is expected that they would conform to the group regardless of whether observers are present or not.

### 2.1 Method

#### 2.1.1 Participants

Participants were 30 3-year-old Korean children (age range = 36.1 months to 49.1 months; mean age = 41.6 months; 13 girls). An additional eight children were tested but excluded from the data analysis due to inattentiveness (1), responding with “I do not know” in a test trial (1), failure to complete the experiment (1), providing inaccurate responses in pretest trials (4), and experimental error (1). The sample size was determined using the G*Power program for independent two groups *t*-test, with the power set at 80% and the significance level at 0.05. This calculation incorporated the Cohen’s *d* effect size (0.97) estimated from the data outlined in Corriveau et al. (2013), the previous study upon which our research was based on.

The children were considered to have given inaccurate responses if they answered incorrectly in three or more of the six pretest trials. The children in this experiment and the subsequent experiments were recruited through the posting of recruitment advertisements on online parenting communities and the distribution of leaflets at a public health center. Informed consent was obtained from the parents of each child. Each child was tested individually in a testing room for approximately 10 min.

#### 2.1.2 Design

We used a between-subjects design, randomly assigning child participants to one of two conditions. In the experimental condition, the children watched scenes on a monitor and answered the experimenter’s questions while two female adults (observers) sat nearby and watched the entire procedure. The observers refrained from responding to informants or children throughout the study, maintaining a neutral facial expression while observing the children. In the control condition, the stimuli and procedures were identical to those in the experimental condition, with the exception that no observers were present during the procedure.

#### 2.1.3 Procedure

Upon arrival, the children engaged in a warm-up with the female experimenter in a waiting room while the children’s parents filled out a brief information sheet and signed the consent form. Once the children seemed comfortable with the laboratory environment and were ready to enter the testing room, they were told that they were going to be shown some pictures and would then need to answer the experimenter’s questions. In the experimental condition, once the child entered the testing room with the experimenter, s/he was greeted by two observers. The child sat on the left side of the experimenter and on the right side of the observers. A computer monitor was placed on the table in front of the child.

In the pretest phase, the children were presented with a set of six pictures depicting prosocial behaviors such as helping, sharing, and comforting. Then the children were asked whether or not the behaviors were acceptable (e.g., “This child is sharing his toy with another child. Is this behavior okay or not okay?”).

**Figure 1:**
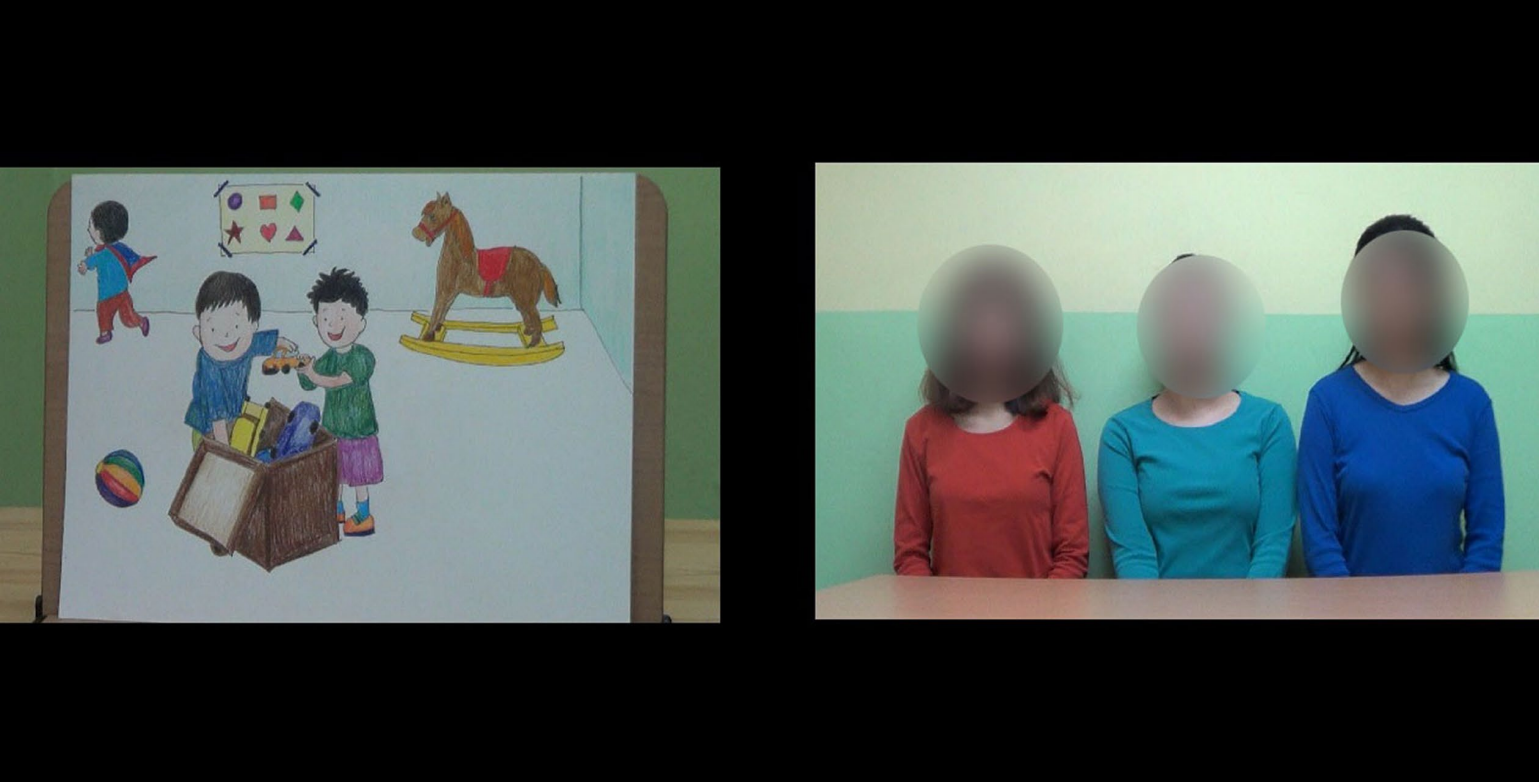
An example of video scenes presented during the test phase in Experiments 1 and 2. Three informants (on the right side) stated whether the behavior shown in picture (on the left side) was OK or not.

The children were then informed that they would watch a video. The experimenter pointed to a static video frame displaying three adult informants and said, “Look! One woman has a red shirt, one has a green shirt, and one has a blue shirt” (see Figure 1). Furthermore, the children were told that the video would show these people’s thoughts on the behaviors shown in the pretest phase and that the children would answer the experimenter’s questions after watching the video.

During the following test phase, children received 6 trials consisting of 4 experimental and 2 filler trials, presented in a fixed order. A constraint was applied, including the filler trials in the first and fourth trials, mirroring Asch’s (1951) seminal study of conformity, in which he sought to enhance the reliability of informants by instructing them to provide the correct answer in the first and intermediate trials. In each trial, children watched video scenes in which the three informants saw each of the pictures shown in the pretest phase and unanimously claimed that the behaviors were “okay” (filler trials) or “not okay” (experimental trials). After hearing the informants’ responses, the experimenter said to the child, “They said this behavior was okay (or not okay). Do you think this behavior is okay or not okay?”

Thus, the children were faced with the dilemma of whether to conform to the unanimous opinion of the three informants, who were regarded as the majority, or to ignore their unanimous opinions. The behavior of interest was whether the children would change their answers after hearing the informants’ opinions. The children’s responses were videotaped and recorded by the experimenter. At the conclusion of the experiment, the experimenter rectified mistaken opinions expressed by the informants (e.g., “They stated that this behavior was not acceptable. However, I believe they are mistaken. It’s possible that there was an error. This behavior is indeed acceptable. What are your thoughts on this behavior? Do you consider it acceptable or not?”). Children who provided the correct response were rewarded with stickers. This procedure aimed to mitigate any potential moral confusion that could have arisen during the experiment.

### 2.2 Results and discussion

Following the procedures of previous studies of preschoolers’ conformity (Corriveau et al., 2009, 2013; Kim et al., 2016), the main dependent variable, conformity, was a dichotomous variable indicating whether the participant conformed at least once out of 3 experimental test trials. Children’s conformity was determined by assessing whether the children’s responses changed after viewing the test video in comparison to their responses during the pretest phase. Data from one experimental test trial which involved a scenario in which a child comforted his/her friend who fell and got hurt was excluded from the final analysis, because 40% of the children (12 out of 30) judged during the pretest phase that engaging in such prosocial behavior was “not okay.” Notably, the children did not exhibit such responses in any of the pretest trials involving other prosocial actions such as helping or sharing. The low percentage of correct judgments about comforting actions could be attributed to the characters’ facial expressions in the picture used for scenarios involving comforting actions, which were more negative compared to the other trials. Consequently, children’s conformity was assessed based on responses in the remaining 3 experimental trials that were included in the analyses. Inspection of the test data indicates that children’s conformity responses were consistent across the three experimental test trials in both conditions.

**Figure 2:**
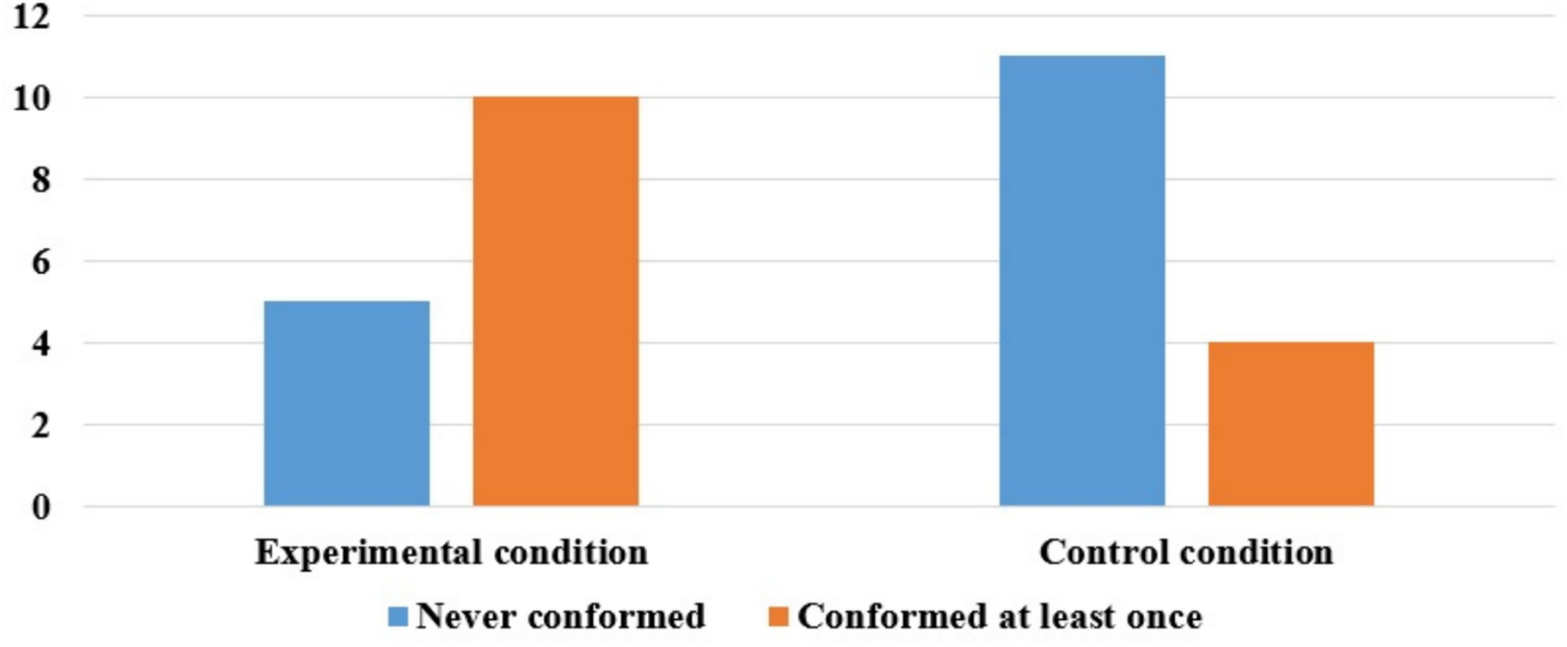
Number of children who conformed or never conformed in the experimental and control conditions, Experiment 1.

Preliminary analyses of the test data revealed no main effect of children’s gender [*F*(1, 28) = 0.00, *p* = 0.962], the data were therefore collapsed across gender in subsequent analyses. We analyzed whether conformity responses varied across participants and across trials based on conditions, and all results yielded statistical significance. Children who reported incorrect answers in one or more trials accounted for 67% (10 out of 15) and 27% (4 out of 15) of the experimental and control conditions, respectively (see Figure 2). A chi-square test of independence was conducted to examine the relation between presence of observers and the children’s conformity behavior. The analysis revealed a significant association, *X*^2^ (1, *N* = 30) = 4.82, *p* = 0.028, indicating that children were significantly more inclined to conform when observers were present. The effect size, as measured by Phi coefficient, was ϕ = 0.40, indicating a moderate effect. Trials which children reported incorrect answers in one or more trials accounted for 53% (24 out of 45) and 20% (9 out of 45) of the experimental and control conditions, respectively. A chi-square test of independence was conducted to examine the relation between presence of observers and the number of trials in which children conformed. The analysis revealed a significant association, *X*^2^ (1, *N* = 90) = 10.77, *p* = 0.001, supporting the previous results. In Experimental condition, children’s conformity responses were observed in 24 out of a total of 45 trial cases, showing no significant difference from chance-level performance (*p* from binomial test = 0.766). In contrast, in the control condition, children seldom conformed, with only 9 occurrences out of a total of 45 trials, less frequently than expected by chance (*p* from binomial test < 0.001).

The results for the control condition are consistent with previous findings that children are resistant to incorrect opinions expressed by the majority (Schillaci and Kelemen, 2014). Preschoolers are very competent at identifying accurate versus inaccurate informants (Koenig et al., 2004), and preschoolers followed the majority’s opinion when the opinions of the majority and a lone dissenter were equally plausible but rejected the majority opinion when it was implausible (Schillaci and Kelemen, 2014). However, the current results suggest that 3-year-olds, despite their ability to judge that morally right behaviors were acceptable, tend to adjust their opinions when watched by adults.

Why did the children conform to the majority’s incorrect opinions when there were observers? It remained unclear whether the mere presence of observers or the possibility to be evaluated by observers promoted children’s conformity behavior. Experiment 2 examined this question.

## 3 Experiment 2

To explore the nature of the observer effect shown in Experiment 1, Experiment 2 implemented the manipulation used by Haun and Tomasello (2011), where children tend to conform more to the group’s incorrect information in public situations where their opinions can be heard by others, even those who cannot see the children, as compared to situations where their opinions cannot be heard by others. There were two conditions: in the headset-on condition, the two observers were wearing headsets that prevented them from hearing the opinions of the children and informants, while in the headset-off condition, the headsets were hanging around their necks, allowing them to hear.

We hypothesized that if children are primarily sensitive to the mere presence of the observers their reactions would be similar in both conditions. However, if potential evaluations by observers who can hear and judge their opinions influence children’s conformity, we predicted that they would demonstrate higher levels of conformity when the observers wore headsets around their necks, compared to the condition where the observers themselves wore the headset.

### 3.1 Method

#### 3.1.1 Participants

Participants were 30 3-year-old Korean children (age range = 36.2 months to 46.5 months; mean age = 41.7 months; 17 girls). An additional nine children were tested but excluded from the data analysis because of failure to complete the experiment (2), inaccurate responses in pretest trials (2), inattentiveness (3), refusal to participate (1), and experimental error (1).

#### 3.1.2 Design and procedure

The stimuli and procedures were identical to those used in Experiment 1, with the following exceptions. In the headset-on condition, the children were watched by observers wearing headsets, whereas in the headset-off condition, the children were watched by observers with headsets hanging around their necks. Prior to the experiment, children were shown the headset, told that the headset was for listening to music, and shown how to use the headset. At the end of the explanation in the waiting room, it was demonstrated that the observers could not hear the child when they were wearing the headsets but could hear the child when the headsets were hanging around their necks. Before the experiment, the child was asked if observers could hear the sound. The majority of children (29 out of 30 children included in the analysis) answered the question correctly, demonstrating a general understanding of how headsets could impact observers’ hearing. The results of the data analysis remained consistent regardless of whether the child who incorrectly answered the question before the experiment was included in the analysis. Consequently, the data from all 30 children, including the one mentioned, were included in the final dataset for analysis.

### 3.2 Results and discussion

Data from one experimental test trial which involved a scenario in which a child comforted his/her friend who fell and got hurt was excluded from the final analysis, because 43% of the children (13 out of 30) judged during the pretest phase that engaging in such prosocial behavior was “not okay” as in Experiment 1. Thus, the data from the remaining 3 experimental test trials were included in the analysis.

**Figure 3:**
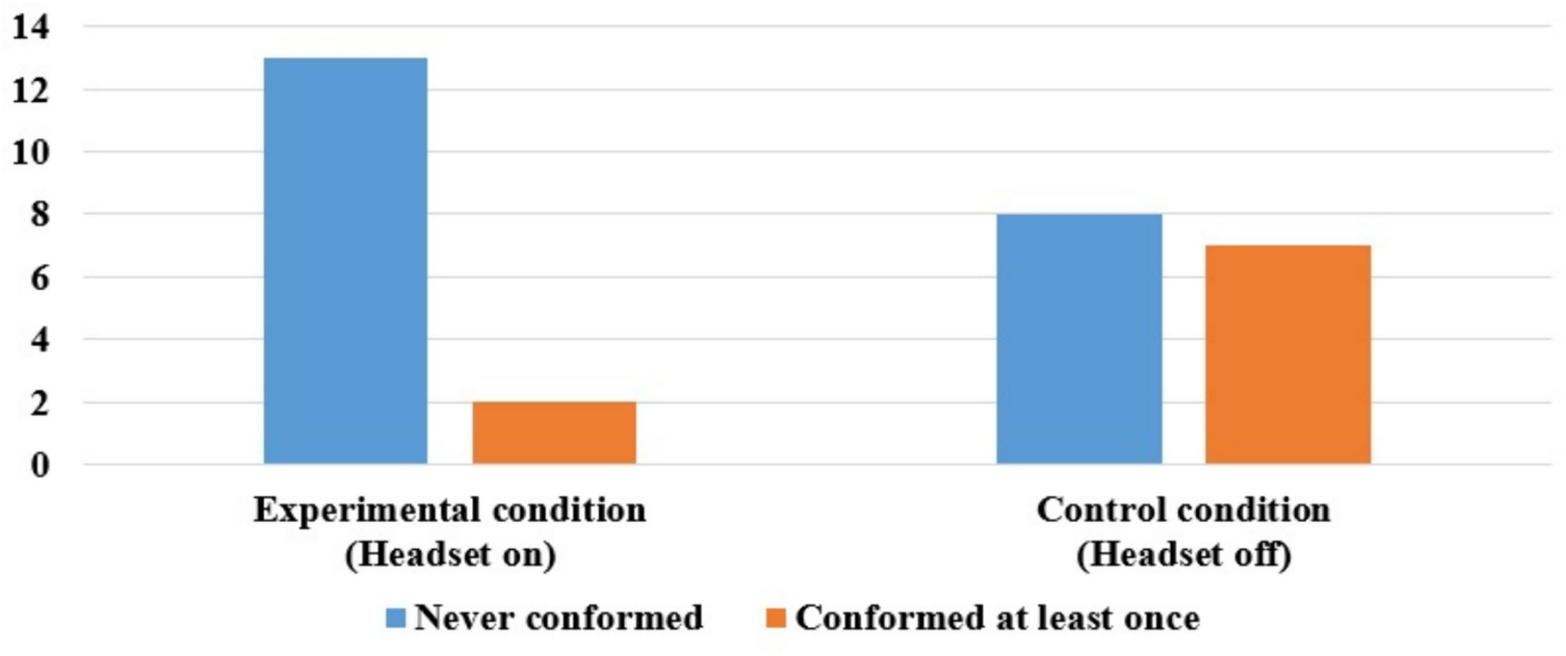
Number of children who conformed or never conformed in the experimental and control conditions, Experiment 2.

Preliminary analyses of the test data revealed no main effect of children’s gender [*F*(1, 28) = 0.50, *p* = 0.486], the data were therefore collapsed across gender in subsequent analyses. We conducted analyses to determine whether there existed variations in conformity across participants and across trials by condition, and all findings showed statistical significance. Children who conformed in one or more trials accounted for 13% (2 out of 15) and 47% (7 out of 15) of the experimental and control conditions, respectively (see Figure 3). There was a significant relationship between observers’ listening availability and the children’s conformity behavior, *X*^2^ (1, *N* = 30) = 3.97, *p* = 0.046. The effect size, as measured by Phi coefficient, was ϕ = 0.36, indicating a moderate effect. Trials which children reported incorrect answers in one or more trials accounted for 7% (3 out of 45) and 40% (18 out of 45) of the experimental and control conditions, respectively. A chi-square test of independence was conducted to examine the relation between whether observers could hear and the number of trials in which children conformed. The analysis revealed a significant association, *X*^2^ (1, *N* = 90) = 13.98, *p* < 0.001, supporting the previous results. When observers wear headsets, children make correct moral judgments significantly above chance, *p* < 0.001.

The results indicated that children were more inclined to align with a group holding morally incorrect opinions when their responses were audible to observers, as opposed to scenarios where their responses were not heard by those observing. Thus, the observer effect observed in Experiment 1 may not be solely attributed to the mere presence of others. Instead, the findings are consistent with the possibility that the observer effect could have been driven by the social pressure exerted by individuals capable of evaluating the participants.

## 4 General discussion

In the present research, we investigated the impact of observer effects on conformity in children’s moral judgments. The findings from Experiments 1 and 2 suggest that although children positively rated prosocial actions in the pretest trial, they rated such actions more negatively after witnessing three adult informants claim that the actions were “not okay,” particularly, in the presence of observers. A notable finding was that the opinions of informants affected the children’s moral judgment behavior, even when they were in separate spaces and thus would be unable to witness children’s moral judgment. This effect was further intensified in the presence of passive observers who neither provided any opinions nor explicitly approved or disapproved children’s behaviors.

In Experiment 2, children displayed higher levels of conformity when there were observers present who could hear their opinions, as opposed to those who could not. These findings suggest that the influence on children’s conformity behavior is not solely attributed to the mere presence of observers, but rather to specific characteristics of the observers, such as their ability to hear the opinions of both the informants and the children. Various processes may be at play in explaining this phenomenon.

First, the children might have interpreted neutral reactions by observers who could hear the informants’ opinions as implicit agreement with observers’ opinions. The perception of implicit approval by observers could have prompted children to perceive a stronger consensus within the group, which in turn may have influenced them to conform more frequently to the informants’ incorrect opinion. Preschoolers are sensitive to the level of group consensus when determining whether to agree with others’ statements (Corriveau et al., 2009; Chen et al., 2013). To examine this possibility in a future study, we could inquire about the children’s “perceptions of the observers” likely attitudes regarding the informants’ opinions. Furthermore, resemblance between the observers and informants such as the same gender, similar age, the same race, might have heightened children’s perception of affiliation and agreement between the two groups. To test this possibility, future research could be conducted with informants and observers having diverse demographic information.

Second, the children might have inferred that observers who could hear their opinions may evaluate them. Observers, by promoting social evaluation, generate an increased interest or consideration of social consequences for individuals (Engelmann et al., 2016). Children might have attempted to present themselves favorably, aiming to appear “good” in the presence of evaluating observers. The children in the present research likely perceived that it would be more desirable to respond similarly to informants and that their judgments would be known to the observers. Children tend to modify their behaviors in order to conform to groups, especially in public settings, with the goal of being perceived as similar to others (Haun et al., 2014). The ability to tailor an individual’s behavior according to an observer may be an indicator of complete strategic reputation management (Fu and Lee, 2007; Watling and Banerjee, 2007; Engelmann et al., 2013; Heyman et al., 2016). The current findings provide additional evidence that children under the age of 5 can manage their reputations (Engelmann and Rapp, 2018; Zhao et al., 2018; Ma et al., 2020). The possibility that children’s conformity to the group might have been driven by social similarity motivation or reputation management strategy suggests that children’s theory of mind may play a critical role in children’s conformity behavior. Exploring how children perceive and anticipate others’ evaluations of them could provide valuable insights into the mechanisms underlying their reactions to others’ opinions. This could serve as an intriguing avenue for future research.

In the current research, when there were no observers, children showed resistance to the group’s morally incorrect judgments. Thus, under conditions of relatively low social pressure, children exhibited the ability to autonomously make subtle and correct judgments without being swayed by conflicting opinions offered by groups. These results contrast with those obtained by Li et al. (2019), in which children conformed to the group’s judgments after listening to the informants’ opinion when making moral judgments. The observed discrepancy can be attributed to the fact that the current study focused on explicit prosocial behaviors, while Li et al. utilized unfamiliar words (e.g., “mib”) to describe ambiguous moral transgressions. The lack of detailed information regarding the specific content of the unfamiliar behavior used by Li et al. (2019) could have contributed to the children’s receptiveness to the opinions of the adult informants in their study.

The study’s exclusive focus on 3-year-old children raises questions about whether the influence of observers on children’s conformity behaviors changes or persists as they grow older. The tendency to alter behaviors in line with the majority and sensitivity to being watched by others are evident throughout development, spanning from childhood to adulthood (Yazdi et al., 2020). Concurrently, various findings suggest potential developmental changes in such inclinations. While the tendency to behave in line with the majority may often become less pronounced over the course of development (Schillaci and Kelemen, 2014; Flynn et al., 2018; Li et al., 2019), reputational concerns emerge as early as 3 years of age (Ma et al., 2020; Asaba and Gweon, 2022) and persist or evolve into more sophisticated forms, possibly with the advance of cognitive skills necessary for reasoning about how others’ perceptions of us, toward adulthood (e.g., Engelmann and Rapp, 2018). Consequently, comprehensive future studies are needed to explore the varying degrees to which being watching by others influences children’s conformity at different developmental stages, which would reveal the changes in the reasons underlying children’s conformity behavior in their developmental context.

The passive observers in our current research were adults, raising a question about the generalizability of the observer effect. Does the act of an adult labeling a behavior as “not okay” possess a unique influence, making children more prone to conform? Would this effect manifest differently if the observers were peers rather than adults? To our knowledge, there is no existing evidence to address this question, but we can speculate that the age of observers might not significantly alter the observed effect, as suggested by previous research. Corriveau et al. (2013) established that the age of informants, whether adults or peers, had no substantial impact on children’s conformity. This conclusion has been further supported by a recent study that echoed similar findings (Li et al., 2021).

There are several limitations we must acknowledge in the current research. First, the sample size was relatively small, and the subjects were composed of Korean children only. Despite the statistically significant differences observed between conditions in this study, further studies involving a bigger sample which consists of a more diverse participant pool are required to determine whether these findings hold across different cultural contexts. Conformity behaviors can manifest differently and be evaluated in different ways by others according to the prevailing cultural values (Clegg et al., 2017). Specifically, culture does not affect children’s ability to judge whether a particular behavior is morally right or wrong but how well children conform to an adult’s counterintuitive assertions in a particular moral situation (Li et al., 2019). To explore and understand the cultural diversity of conformity behavior, which is the result of the developmental process (Berg and Bass, 1961), it is necessary to preferentially determine when the sensitivity to social pressure that provokes conformity behavior emerges.

Second, the study did not employ counterbalancing or randomization of trial order. Similar to Asch’s (1951) experiment, our research presented stimuli in a consistent order to all participants. Nevertheless, considering that our sample size was significantly smaller than Asch’s, a more meticulous approach in presenting stimuli appears necessary to validate the findings.

The current research has significant implications for understanding the social contextual factors that influence the development of children’s moral judgment. While the influence of social pressure, such as the presence of individuals expressing certain opinions, has been extensively studied, there is limited evidence regarding the impact of the mere presence of passive observers who neither express opinions nor directly instruct children in any particular manner. Our findings provide initial evidence that children as young as 3 years old are susceptible to perceived social pressure, which may be driven by the passive presence of others observing them during moral judgment. It would have been valuable to measure the confidence children have in their judgment after aligning their opinions with others. If they maintain their assessment that the prosocial actions were “not okay,” it may suggest more than social pressure affecting children’s behavior. Future research can examine this possibility.

In summary, this research highlights the significance of social context in shaping children’s moral judgment, shedding light on the role of passive observers and the impact of perceived social pressure. However, it is important to note that the young participants in our study did not uniformly conform to “group consensus” when no observers were present, demonstrating independence in their judgment. Further exploration of factors that could potentially impede children’s ability to make autonomous moral judgments, especially under the sway of social influence, can provide deeper insights into the development of moral reasoning in children and inform interventions aimed at promoting ethical decision-making.

## Data availability statement

The original contributions presented in the study are included in the article/supplementary material, further inquiries can be directed to the corresponding author.

## Ethics statement

The studies involving humans were approved by Yonsei University Institutional Review Board. The studies were conducted in accordance with the local legislation and institutional requirements. Written informed consent for participation in this study was provided by the participants’ legal guardians/next of kin.

## Author contributions

YL: Conceptualization, Data curation, Investigation, Methodology, Project administration, Writing – original draft, Writing – review & editing. HS: Conceptualization, Funding acquisition, Supervision, Writing – review & editing.
